# A New Work on Anatomy

**Published:** 1853-09

**Authors:** 


### A NEW WORK ON ANATOMY

A work on Anatomy will soon be issued by Lippincott, Grambo and Co., from the pen of Tobias G. Richardson, M. D., Demonstrator o Anatomy in the University of Louisville. From the labor bestowed upon it, and the complete mastery of his subject possessed by the author we look for a work of uncommon accuracy and excellence.

